# A Study on the Mechanical and Wear-Resistance Properties of Hybrid Fiber Mortar Composites with Low Water–Cement Ratios

**DOI:** 10.3390/ma17153798

**Published:** 2024-08-01

**Authors:** Shuangxi Li, Zimin Dang, Chunmeng Jiang, Xinguang Xia

**Affiliations:** 1 College of Hydraulic and Civil Engineering, Xinjiang Agricultural University, Urumqi 830052, China; dangzimin@163.com (Z.D.); jiangcm93@163.com (C.J.); 2 Xinjiang Key Laboratory of Hydraulic Engineering Security and Water Disasters Prevention, Urumqi 830052, China; 3 Xinjiang Remote Measurement Engineering Technology Co., Ltd., Changji 831100, China

**Keywords:** aramid fiber, calcium sulfate whisker, basalt fiber, mortar composites with a low water–cement ratio, microstructure

## Abstract

Based on mortar composites with a low water–cement ratio, the effects of hybrid aramid fiber (AF), calcium sulfate whisker (CSW), and basalt fiber (BF) on their mechanical properties and wear resistance were studied, and the correlation between wear resistance and compressive strength are discussed. A microstructure analysis was conducted through scanning electron microscopy (SEM) and the nitrogen-adsorption method (BET). The research results show that compared with the control group, the compressive strength, flexural strength, and wear resistance of the hybrid AF, CSW, and BF mortar composites with a low water–cement ratio increased by up to 33.6%, 32%, and 40.8%, respectively; there is a certain linear trend between wear resistance and compressive strength, but the discreteness is large. The microstructure analysis shows that CSW, AF, and BF mainly dissipate energy through bonding, friction, mechanical interlocking with the mortar matrix, and their own pull out and fracture, thereby enhancing and toughening the mortar. A single doping of CSW and co-doping of CSW and AF can refine the pore structure of the mortar, making the mortar structure more compact.

## 1. Introduction

The pavement of highways and airport runways is often subjected to the wear and tear from vehicle and airplane tires. Therefore, cement-based materials in these areas need to have high wear resistance [1,2]. Currently, the primary methods to enhance the wear resistance of cement-based materials include reducing the water-to-cement ratio and incorporating a large amount of active mineral admixtures. However, lowering the water-to-cement ratio increases the brittleness of the material, making it more prone to shrinkage and cracking. Studies have shown that incorporating fibers into cement-based materials can enhance their toughness and reduce shrinkage cracking [3,4,5].

Commonly used fibers include steel fibers, polypropylene fibers, basalt fibers, and aramid fibers. Song et al. found that incorporating a 1.5%-by-volume admixture of steel fibers can increase the compressive strength of concrete by 15.3% [6]. However, steel fibers have disadvantages such as a susceptibility to corrosion and rust, high cost, and high production energy consumption [7]. Polypropylene fibers have a lower tensile strength and elastic modulus but can reduce shrinkage and cracking in concrete [8]. Banthia et al. found that coarse polypropylene fibers are more effective than fine polypropylene fibers in controlling the plastic shrinkage cracking of concrete [9].

Basalt fibers (BFs) have advantages such as high tensile strength, high elastic modulus, corrosion resistance, and low cost. They are lighter, cheaper, and have a lower production energy consumption compared to steel fibers [10,11,12]. Compared to polypropylene fibers, BF has higher strength and a higher elastic modulus. However, incorporating BF can lead to a decrease in the compressive strength of concrete [13,14]. Jiang et al. found that when a 0.3%-by-volume admixture of BF is incorporated into concrete, its flexural strength and tensile strength reach optimal levels [15]. Kabay’s research indicates that BF can enhance the wear resistance of concrete [16].

Aramid fibers (AFs) are a new type of high-performance synthetic organic fiber. Their tensile strength is five times that of steel fibers, and their elastic modulus is twice that of steel wires. They are lightweight, corrosion-resistant, and wear-resistant, but their application in cement-based materials has been relatively recent [17,18]. Incorporating AF can improve the mechanical properties and impact resistance of concrete. However, AF is relatively expensive [19,20]. Wang et al.’s research shows that incorporating 24 mm of AF at 10% of the cement mass can significantly improve the impact resistance of concrete [21]. Aramid fibers not only have higher strength than other synthetic and natural fibers but are also more durable in the alkaline environment of concrete compared to steel fibers. Existing research on aramid fiber cement-based materials is limited, especially regarding their wear resistance. Therefore, further research on aramid fiber cement-based materials is necessary.

Calcium sulfate whiskers (CSWs) are sub-nanometer fibers made from gypsum. They have high strength, wear resistance and corrosion resistance and are low-cost, with wide applications [22,23,24,25]. Cao et al. found that an appropriate amount of CSW can effectively refine the pore structure inside concrete [26]. CSW is more adaptable to the alkaline environment of concrete compared to steel fibers and is often used as a filler in friction materials. However, it may lead to a decrease in the early strength of concrete [27,28]. In the later stages of concrete, undissolved CSW continues to dissolve, which may cause sulfate attack damage to the concrete [29].

Different types of fibers have their own properties. Mixing multiple fibers into cement-based materials can complement each other’s advantages, making it more reasonable than using a single type of fiber alone [30,31,32]. In recent years, many scholars have focused their research on hybrid fiber cement-based materials [33]. Current research indicates that hybrid fibers can better enhance the mechanical and wear-resistance properties of concrete compared to single fibers [34,35,36,37]. Zhang et al. found that mixing CSW and BF can effectively reduce the gas permeability of concrete [38]. Chen et al.’s research shows that when a 0.15% basalt fiber and 0.11% polyacrylonitrile fiber are incorporated into concrete, its mechanical properties are the best [39].

Feng et al. studied the effects of different lengths of aramid fibers on the mechanical and impact-resistance properties of cement-based materials. The results show that 6 mm aramid fibers had the best effect [40]. Currently, researchers often use 12 mm basalt fibers, because they can enhance the mechanical properties of concrete [16]. The market-produced calcium sulfate whiskers range in length from 30 to 150 µm, with a diameter of about 1–4 µm. There is limited research on the effects of different lengths of calcium sulfate whiskers on the properties of concrete. Therefore, this study decided to incorporate 6 mm aramid fibers, 12 mm basalt fibers, and 10–200 micrometer calcium sulfate whiskers into cement-based materials for performance testing.

Currently, there is extensive research on the mechanical and durability properties of ordinary cement-based materials with a single AF, CSW, or BF incorporation. However, there is limited research on the mechanical and wear-resistance properties of cement mortar composites with a low water-to-cement ratio and with a mixed AF, CSW, and BF incorporation. Therefore, this paper studies the effects of a mixed incorporation of AF, CSW, and BF on the mechanical and wear-resistance properties of cement mortar composites with a low water-to-cement ratio. It discusses the correlation between wear resistance and compressive strength and analyzes the fiber reinforcement mechanism from a microscopic perspective using scanning electron microscopy (SEM) and nitrogen-adsorption tests (BETs). This has certain reference significance for the application and improvement of the theoretical system of mixing AF, CSW, and BF in cement-based materials.

## 2. Materials and Methods

### 2.1. Main Test Materials

The chemical compositions of cement, silica fume, and fly ash were analyzed using X-ray fluorescence, and the results are shown in Table 1.

The cement (P.O 42.5R Ordinary Portland Cement) had compressive strengths of 27.3 MPa and 42.5 MPa at 3 days and 28 days, respectively. The fly ash (Grade I Fly Ash) had a fineness of 8.2%. The silica fume had an SiO_2_ content of 88%.

The elemental composition of the aramid fiber, basalt fiber, and calcium sulfate whiskers was analyzed using Energy-Dispersive Spectroscopy (EDS), and the results are shown in Table 2.

**Figure 1:**
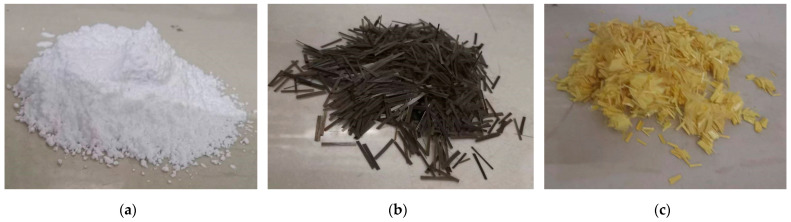
Different appearances of various fibers: (**a**) calcium sulfate whiskers; (**b**) basalt fiber; (**c**) aramid fiber.

Calcium sulfate whiskers (CSWs) are needle-like anhydrous calcium sulfate whiskers. aramid fiber (AF) is chopped para-aramid fiber. basalt fiber (BF) is chopped basalt fiber. Figure 1 shows images of the calcium sulfate whiskers, basalt fiber, and aramid fiber. Table 3 presents the physical properties of the calcium sulfate whiskers, and Table 4 presents the physical properties of the aramid fiber and basalt fiber.

Fine aggregate, continuously graded natural medium sand, with a fineness modulus of 2.97, an apparent density of 2620 kg/m^3^, and a bulk density of 1531 kg/m^3^, was used. An admixture, with a rapid-dissolving polycarboxylate superplasticizer and a water-reduction rate of 45%, was used, as was domestic water from Urumqi City, in compliance with water-testing regulations.

### 2.2. Mix Design for the Experiment

According to “Reactive Powder Concrete” (GB/T 31387-2015), the mix design for the mortar control group was conducted [41]. The mixed proportions for the mortar control group are shown in Table 5.

AF and BF were added to the mortar separately at 0.1% of the sample volume or were simultaneously added to the mortar at 0.1% of the sample volume, with volume mixing ratios of 2:1, 1:1, and 1:2; CSW was added to the mortar at 1%, 2%, 3%, and 4% of the mass of the cementitious material; CSW was added to the mortar at 1%, 2%, 3%, and 4% of the mass of the cementitious material, and 0.1% of the sample volume of AF and BF was added, respectively; CSW was incorporated into the mortar at 1%, 2%, 3%, and 4% of the mass of the cementitious materials, and an AF and BF combination was added (with the total incorporation amount being 0.1% of the specimen volume, with volume mix ratios of 2:1, 1:1, and 1:2, respectively). The experimental scheme for the hybrid fiber cement mortar composites with a low water-to-binder ratio is shown in Table 6.

### 2.3. Sample Preparation

According to the mix ratio in Table 3 and the test scheme in Table 4, the steps for specimen preparation were as follows: (1) weigh the cement, silica fume, water-reducing agent, and fly ash and put them into the mixer in sequence, and dry mix at a low speed for 1 min; (2) pour the water and the uniformly mixed calcium sulfate whisker solution into the mixer, and mix at a low speed for 1 min; (3) pour the natural sand and basalt fiber uniformly into the mixer, mix at a low speed for 1 min first, then mix at a high speed for 1 min and 30 s; and (4) pour the aramid fiber uniformly into the mixer, and mix at a high speed for 3 min to obtain the freshly mixed material. The mixed material was loaded into molds, and the molds were moved to a vibrating table for compaction and leveling and were covered with a plastic wrap. Standard curing was performed for 1 day before demolding, and then the mixed material was placed in a standard curing room for curing until the test age was reached.

### 2.4. Test Methods

#### 2.4.1. Mechanical Performance Testing

##### Flexural and Compressive Strength Tests

The flexural and compressive strength tests of the mortar prism specimens were conducted according to the “Test Method for Strength of Cement Mortar” (GB/T 17671-2021) [42]. An electro-hydraulic flexural and compressive testing machine (TYA300BI type) was used to test the flexural and compressive strengths of 28-day prism specimens. The loading speed for the flexural strength test of the prism specimens was set to 50 N/s, and the loading speed for the compressive strength test of the broken specimens was set to 2.4 KN/s. Three 40 mm × 40 mm × 160 mm prism specimens were made for each group. The flexural strength was the average result of three flexural tests for each group of prismatic specimens, and the compressive strength was the average result of six compressive strength tests for each group of specimens after the flexural test, with a numerical error controlled within 15%. The formula for calculating flexural strength is shown in Equation (1), and the formula for calculating compressive strength is shown in Equation (2).

(1) $$f_{\;cc}=\frac{3PL}{2bh^{2}}=0.00234P$$

(2) $$f_{cu}=\frac{P}{A}=0.625P$$

In Equation (1), *f_cc_* is the flexural strength of the specimen (MPa); *P* is the failure load of the specimen (N); *L* is the span length between supports, which is 100 mm; and *b* and *h* are the side length and height of the specimen cross-section, both of which are 40 mm.

In Equation (2), *f_cu_* is the compressive strength of the specimen (MPa), *P* is the failure load of the specimen (KN), and A is the compression area (mm^2^), which is 40 mm × 40 mm.

#### 2.4.2. Abrasion-Resistance Test

**Figure 2:**
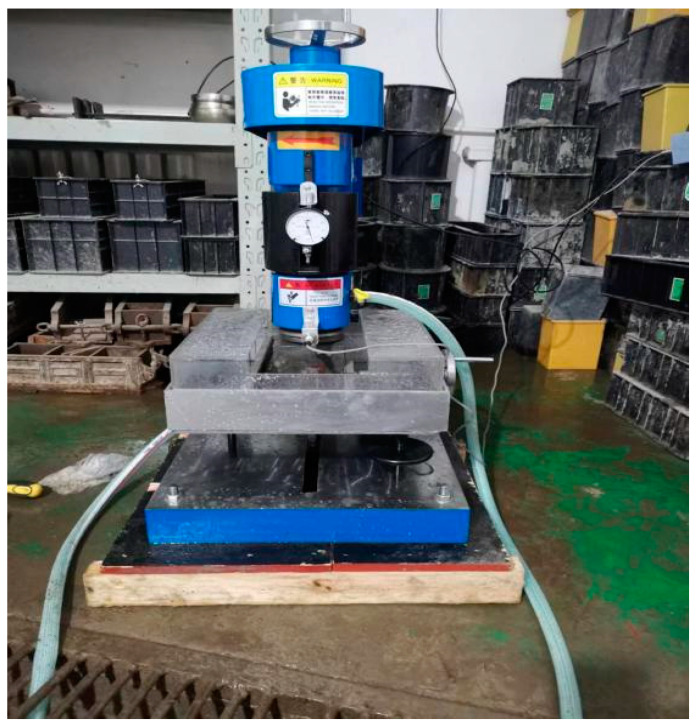
Concrete ball bearing wear-resistant machine.

The abrasion-resistance test of the mortar cube specimens was conducted according to the “Test Method for Abrasion Resistance of Concrete and Its Products” (GB/T 17671-2021) [43]. A concrete ball bearing abrasion machine (model NS-2) was used to test the 28-day cube specimens for abrasion resistance. The specimen was fixed, and the grinding head was pressed onto the side of the specimen. The water source was turned on to submerge the abraded surface of the specimen, and the motor was started. The grinding head was stopped once every 1000 revolutions to measure the depth of the grinding groove with a dial gauge until the grinding head reached 5000 revolutions. The test was then stopped, and the depth of the grinding groove was measured. Each group consisted of five 100 mm × 100 mm × 100 mm cube specimens. After discarding the maximum and minimum values, the average of three measurements was taken as the test result, accurate to 0.01 mm. The abrasion-testing machine is shown in Figure 2.

The wear-resistance index is defined as follows:

(3) $$I_{a}=\frac{\sqrt{R}}{P}$$

In Equation (3), *I_a_* is the index of abrasion resistance, accurate to 0.01; *R* is the number of revolutions of the grinding head, in thousands; and *P* is the depth of the abrasion groove, mm.

#### 2.4.3. Microstructure Test

Using the ZEISS Sigma 300 scanning electron microscope (SEM) produced by Carl Zeiss AG (Jena, Germany) to observe the surface microstructure of AF, CSW, and BF mortar samples. Fragments were taken from the interior of the specimens after failure and made into thin slices of about 1 cm × 1 cm × 1 cm. Hydration was terminated with anhydrous ethanol, and the slices were dried, vacuum-coated with gold, and observed under the scanning electron microscope for the fiber microstructure on the sample surface.

Using the ASAP2460 surface area and porosity analyzer (BET) produced by Micromeritics Instrument Corporation (Norcross, GA, USA) to test the pore structure of 3% CSW mortar and 2% CSW + 0.1% AF mortar. After the sample was destroyed, fragments from the interior were taken, and hydration was terminated with anhydrous ethanol. The fragments were dried and ground into particles smaller than 3 mm, weighing about 120 mg, with vacuum degassing at 40 °C for 24 h, and a low-temperature nitrogen-adsorption test was conducted on the internal pore structure.

## 3. Results and Discussion

### 3.1. Mechanical Properties

#### 3.1.1. Compressive Strength

**Figure 3:**
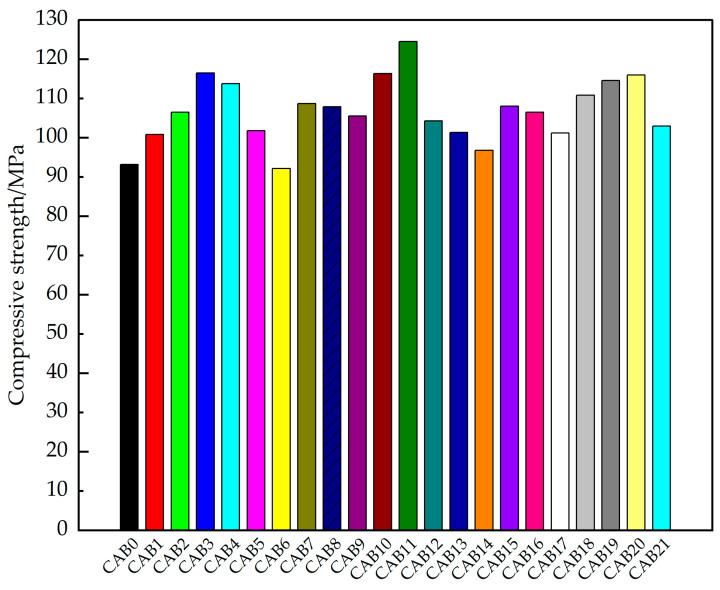
Compressive strength of hybrid fiber-reinforced mortars.

The results of the compressive strength test for the different types of fiber mortars are shown in Figure 3. With an increase in the CSW content, the compressive strength of the mortar first increased and then decreased. The compressive strength of the mortar mixed with 3% CSW (CAB3) increased significantly by 25.0% compared to the control group (CAB0). This is because CSW can not only fill the internal pores of a mortar but can also hydrate to form ettringite to fill the internal pores of a mortar, thereby improving the strength of the mortar [26]. The compressive strength of the mortar mixed with AF alone (CAB5) slightly increased, while the compressive strength of the mortar mixed with BF alone (CAB6) slightly decreased. Compared with the CAB0 group, the compressive strength of the mortar mixed with AF alone increased by 9.2%, and the compressive strength of the mortar mixed with BF alone decreased by 1.1%. This may be because a mortar with a low water-to-binder ratio has a relatively viscous paste, and basalt fibers are sheet fibers bundled by a sizing agent and are slightly longer in length, making it difficult for monofilaments to disperse evenly in the paste, easily causing more weak interfaces in the mortar and leading to a decrease in the mortar strength [44,45]. In Section 3.5, the uneven distribution of basalt fibers is shown in the SEM images.

The compressive strength of the mortar with both AF and BF (CAB7~CAB9) was higher than that of the mortar mixed with AF alone (CAB5), the mortar mixed with BF alone (CAB6), and the control group (CAB0). As the BF content increased, the compressive strength of the mortar with both AF and BF gradually decreased, indicating that AF contributes more to the compressive strength. With an increase in the CSW content, the compressive strength of the mortar with both CSW and AF and CSW and BF (CAB10~CAB17) first increased and then decreased but was still higher than that of the control group (CAB0). Among the mortars with both CSW and AF and CSW and BF, the compressive strength of the mortar with 2% CSW + 0.1% AF (CAB11) was the highest and was 33.6% higher than that of the control group (CAB0).

With an increase in the CSW content, the compressive strength of the mortar with CSW, AF, and BF (CAB18~CAB21) first increased and then decreased, and the compressive strength was higher than that of the control group (CAB0). When the CSW content is too high, mixing CSW, AF, and BF in a mortar may become uneven, causing clumping and leading to a decrease in the mortar strength [46]. Among the mortars with CSW, AF, and BF, the compressive strength of the mortar with 3% CSW + 0.067% AF + 0.033% BF (CAB20) was the highest and was 24.4% higher than the control group (CAB0).

The mortar with 2% CSW + 0.1% AF (CAB11) had the best compressive strength among all the compressive-test specimens. Therefore, we believe that the most suitable fiber ratio for improving the compressive strength is 2% CSW + 0.1% AF. The dual mixing of CSW and AF has the best enhancing effect on the compressive strength of mortars, which is superior to the best enhancing effect of the triple mixing of CSW with different proportions of AF and BF, as well as the best enhancing effect of the single mixing of CSW, AF, and BF on the compressive strength of mortars. The analysis shows that this is because the reinforcing effect of CSWs and AFs of different sizes can effectively suppress the early shrinkage cracking of mortars, can reduce the inherent defects of mortars, and can constrain the microcracks and lateral deformations caused by compressive stress at different scales and stress stages, thereby significantly improving the compressive strength of mortars [47,48,49]. The monofilaments of sheet basalt fibers may be difficult to disperse in a mortar with a low water-to-binder ratio, easily increasing weak interfaces, and their elastic modulus and stiffness are not as good as those of aramid fibers.

Qiao et al. [50] also found that aramid fibers can enhance the compressive strength of cement-based materials with a low water-to-binder ratio. This is similar to the test results of this paper. Koksa et al. [51] studied the mechanical effects of micro-steel fibers and basalt fibers on a silica fume mortar and found that basalt fibers would reduce the compressive strength of the mortar. The finding that basalt fibers have a negative effect on the compressive strength of mortars in this paper is consistent with Koksa’s research results.

#### 3.1.2. Flexural Strength

**Figure 4:**
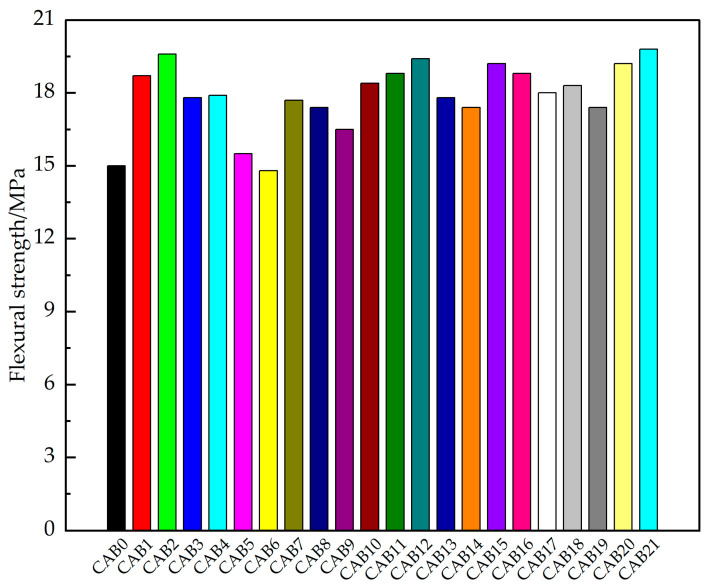
Flexural strength of hybrid fiber-reinforced mortars.

The results of the flexural strength of the different types of fiber mortars are shown in Figure 4. With an increase in the CSW content, the flexural strength of the mortars first increased and then decreased. The flexural strength of the mortar with 2% CSW (CAB2) increased significantly, with an increase of 30.7% compared to the control group (CAB0). The flexural strength of the mortar with only AF (CAB5) slightly increased, while the flexural strength of the mortar with only BF (CAB6) slightly decreased, which is consistent with the compressive strength pattern. Compared with the control group (CAB0), the flexural strength of the mortar with only AF increased by 3.3%, while that with only BF decreased by 1.3%. Zhang [52] found that the optimal CSW content for enhancing mortar strength is 1%, with an increase of 28.41% compared to a benchmark group. In this study, the optimal CSW content is 2%, and the enhancing effect of CSW on the mortar is greater than that found in Zhang’s study. This is because the sand-to-binder ratio in this study is higher than that in Zhang’s study, and the water-to-binder ratio is lower, allowing the cement paste to encapsulate more CSW, and more CSW exerts a toughening effect. The lower water-to-binder ratio enhances the bonding strength between the CSW and mortar, thus enhancing the toughening effect of the CSW.

The flexural strength of both the AF- and BF-mixed mortars (CAB7~CAB9) was higher than that of the mortars with only AF (CAB5), only BF (CAB6), and the control group (CAB0). As the proportion of BF increased, the flexural strength of the mortar gradually decreased, which is consistent with the compressive strength pattern. This indicates that AF is the main contributor to the flexural strength of a mortar. With an increase in the CSW content, the flexural strength of the mortars with both CSW and AF and CSW and BF (CAB10~CAB17) first increased and then decreased, all of which were higher than that of the control group (CAB0), which is consistent with the compressive strength pattern. Among the mortars with both CSW and AF and CSW and BF, the mortar with 3% CSW + 0.1% AF (CAB12) had the highest flexural strength, with an increase of 29.3% compared to the control group (CAB0).

An appropriate amount of triple doping with CSW, AF, and BF improves the flexural strength of a mortar, and the flexural strength of the mortar with CSW, AF, and BF was greater than that of the CAB0 control group. Among the mortars mixed with CSW, AF, and BF, the mortar with 4% CSW + 0.067% AF + 0.033%BF (CAB21) had the highest flexural strength, which was 32% higher compared to the CAB0 control group.

The mortar with 4% CSW + 0.067% AF + 0.033% BF (CAB21) had the best flexural strength among all the flexural-test specimens. Therefore, we believe that the most suitable fiber ratio for improving the flexural strength is 4% CSW + 0.067% AF + 0.033% BF. The analysis reveals that this is related to the flexural failure mode of the specimens. The failure in flexural strength was due to the propagation of a single crack. The CSW, AF, and BF fibers mixed into the mortar provided multi-scale and progressive crack resistances at different stages of crack developments, significantly enhancing the crack-resistance capability. This crack resistance was the strongest, mitigating the negative impact of the poor dispersion of basalt fiber monofilaments on strength [53,54,55]. Jiang et al. [56] found that different contents of a 12 mm basalt fiber can reduce the 28-day flexural strength of a cement mortar, which is similar to the results of this study. Feng et al. [40] found that adding a 12 mm aramid fiber can enhance the flexural strength of cement-based materials, which is similar to the results of this study.

### 3.2. Abrasion Resistance

**Figure 5:**
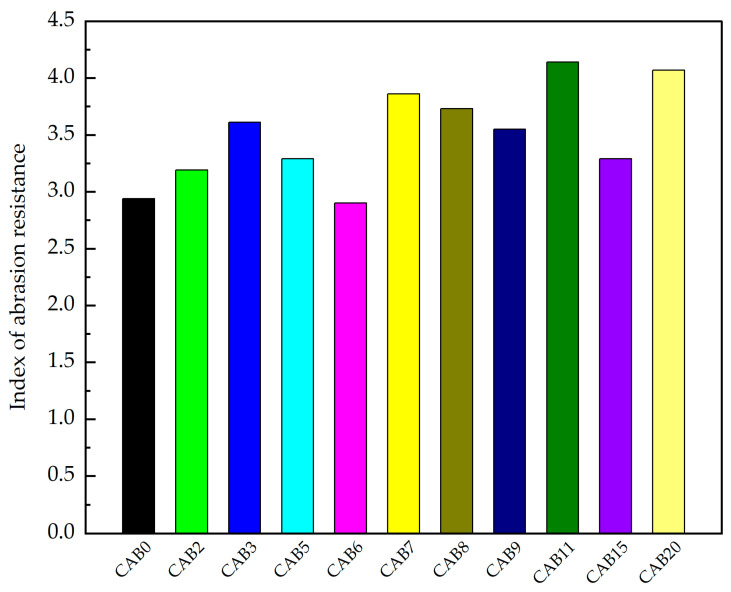
Mixed fiber mortar wear-resistance index.

The results of the abrasion-resistance indices of the different types of fiber mortars are shown in Figure 5. The abrasion-resistance indices of mortars with 2% CSW (CAB2) and 3% CSW (CAB3) were higher than those of the control group (CAB0), with increases of 8.5% and 22.8%, respectively, compared to the control group (CAB0). The abrasion-resistance index of the mortar with only AF (CAB5) was higher than that of the control group (CAB0), while the abrasion-resistance index of the mortar with only BF (CAB6) was slightly lower than that of the control group (CAB0). Compared with the control group (CAB0), the abrasion-resistance index of the mortar with only AF increased by 11.9%, while that with only BF decreased by 1.4%. Grzeszczyk et al. [57] found that adding 2–10 kg/m^3^ of basalt fiber reduces the abrasion resistance of reactive powder concrete, which is similar to the results of this study. Reference [58] indicates that calcium sulfate whiskers may affect the abrasion resistance of cement-based materials.

The abrasion-resistance indices of the AF- and BF-mixed mortars (CAB7~CAB9) were higher than those of mortars with only AF (CAB5), only BF (CAB6), and the control group (CAB0). As the proportion of BF increased, the abrasion-resistance index of the AF- and BF-mixed mortars gradually decreased, indicating that AF is the main contributor to the abrasion-resistance index of mortars. The abrasion-resistance index of the mortar with 0.067% AF + 0.033% BF (CAB7) increased significantly, with an increase of 31.3% compared to the control group (CAB0).

The abrasion-resistance index of the mortar with CSW and AF (CAB11) was higher than that of the mortar with only AF (CAB5) and the control group (CAB0), and the abrasion-resistance index of the mortar with CSW and BF (CAB15) was higher than that of the mortar with only BF (CAB6) and the control group (CAB0). Compared with the control group (CAB0), the abrasion-resistance index of the mortar with 2% CSW + 0.1% AF (CAB11) increased by 40.8%, and that with 2% CSW + 0.1% BF (CAB15) increased by 11.9%.

It can be seen that the wear-resistance index of the mortar with CSW, AF, and BF (CAB20) was greater than that of the mortar with both AF and BF (CAB7) and the control group (CAB0); compared to the control group (CAB0), the wear-resistance index of the mortar with 3% CSW + 0.067% AF + 0.033% BF increased by 38.4%.

The mortar with 2% CSW + 0.1% AF (CAB11) had the highest abrasion-resistance index among all the abrasion-test specimens. Therefore, we believe that the most suitable fiber ratio for improving abrasion resistance is 2% CSW + 0.1% AF. The reason for why the compressive strength of the mortar with 2% CSW + 0.1% AF is the highest among the abrasion-test specimens may be that compressive strength is considered to be an important factor controlling the abrasion resistance of cement-based materials [59]. This is consistent with the discussion in reference [60], which states that abrasion resistance increases with an increase in the concrete compressive strength.

### 3.3. Appearance after Specimen Wear

**Figure 6:**
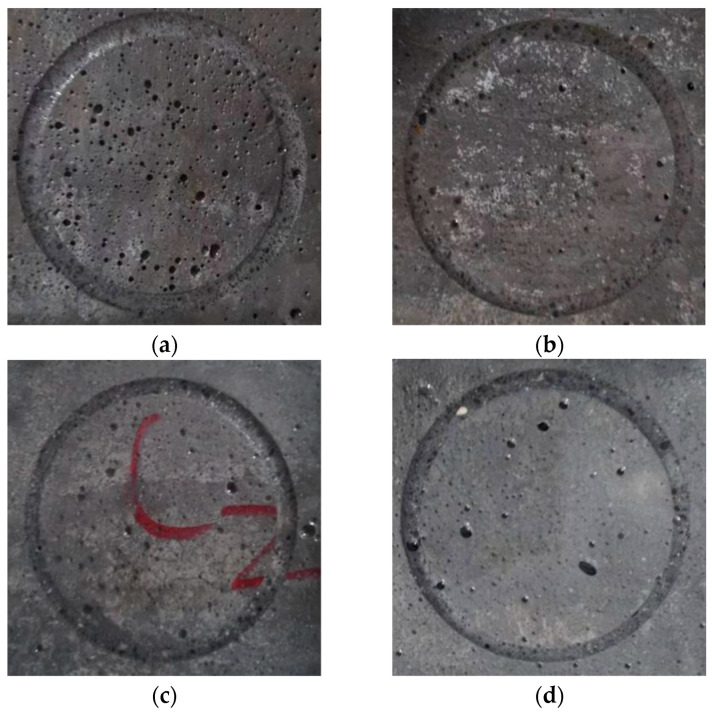
Failure modes of different types of fiber mortars: (**a**) CAB0 control group; (**b**) CAB7 group; (**c**) CAB11 group; (**d**) CAB20 group.

The surface wear patterns of the different types of fiber mortars are shown in Figure 6. From the results of the combinations of CAB0, CAB7, CAB11, and CAB20, it can be observed that the surface wear grooves of the control group specimens without fiber were deeper and wider. When touching the surface of the wear grooves, it felt relatively smooth, whereas the surface wear grooves of the mixed-fiber mortar specimens were reduced in both depth and width. When touching the wear grooves, it felt relatively rough. The analysis shows that the addition of mixed fibers effectively enhances the bonding strength between the paste and the fine aggregate, improving the compactness of the mortar structure, thereby enhancing the wear resistance of the mortar. Therefore, the mixed-fiber mortar shows a significant improvement in wear conditions. As the grinding head compresses and rubs against the specimen surface, the mortar is worn away, exposing the mixed fibers and making the surface rougher, thereby increasing the friction resistance of the hardened paste surface and further enhancing the wear resistance of the mortar.

### 3.4. Relationship between Wear-Resistance Index and Compressive Strength

**Figure 7:**
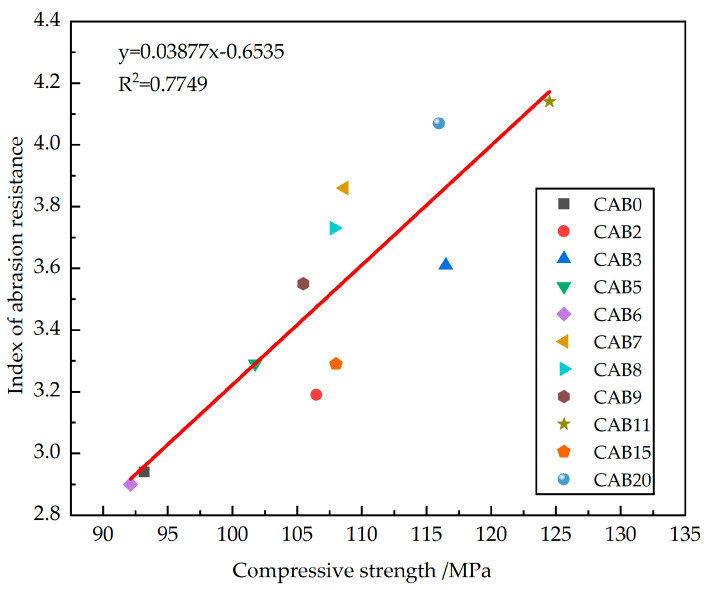
The relationship between the wear-resistance index and compressive strength of mixed-fiber mortars.

As shown in Figure 7, the compressive strength of the different types of fiber mortars exhibited a certain linear trend with their abrasion-resistance indices, but the data are quite scattered. This indicates that the abrasion-resistance indices of different types of fiber mortars are not entirely dependent on their compressive strength. The abrasion-resistance indices of CAB7~CAB9 and CAB20 are above the regression line; the abrasion-resistance indices of CAB5 and CAB6 are basically on the regression line; and the abrasion-resistance indices of CAB2, CAB3, CAB11, and CAB15 are all below the regression line. This shows that the abrasion-resistance indices of different types of fiber mortars do not depend on their compressive strength grade. Therefore, the compressive strength can only be used as a related indicator to characterize the abrasion-resistance indices of different types of fiber mortars, but it is not a decisive parameter.

### 3.5. Microstructure

**Figure 8:**
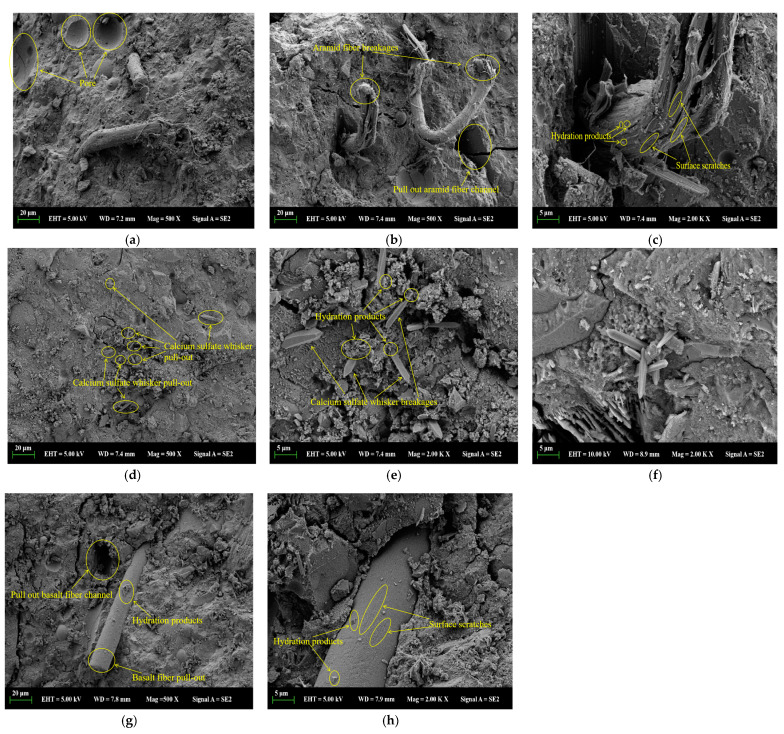
SEM images of different types of fiber mortars. (**a**) Aramid fiber existing forms; (**b**) aramid fiber failure modes; (**c**) aramid fiber surface conditions; (**d**) calcium sulfate whisker distribution; (**e**) calcium sulfate whisker surface conditions; (**f**) calcium sulfate whisker agglomeration phenomena; (**g**) basalt fiber distribution; (**h**) basalt fiber surface conditions.

A scanning electron microscope was used to analyze the microscopic morphological changes of the mortar matrix fractures with the incorporation of AF, CSW, and BF, respectively. From Figure 8a, it can be seen that the flexible AF appeared in a bent or twisted state in the matrix, indicating that AF had a large deformation capacity, enhancing the mechanical bite force with the matrix. Many pores on the fracture surface of the matrix with AF incorporation were observed, which is due to the fact that the matrix with AF is relatively viscous, making it difficult for bubbles to vibrate out, and AF’s hydrophobic surface introduces a large amount of air into the matrix, leading to an increase in the internal pores of the matrix [61].

From Figure 8b, it can be seen that the pulled-out AF and the pits left by its extraction were observed, indicating that AF dissipated energy through pulling out and breaking. As shown in Figure 8c, hydration product particles were observed on the surface of the AF, indicating that the AF had a chemical bonding force with the matrix and good adhesion with the matrix. A flocculent structure on the AF surface was observed, which is due to the frictional tearing marks when AF is pulled out under force, indicating that a relative displacement between AF and the matrix generates frictional force [62,63]. Therefore, AF enhances the macroscopic performance of the matrix through the mechanical bite force, bonding force, frictional force with the matrix, and its own breaking and pull-out energy dissipation.

As shown in Figure 8d, a large quantity of the CSW was well dispersed in the matrix, forming a three-dimensional random distribution network that enhanced the overall stress transfer performance of the matrix. As shown in Figure 8e, hydration product particles were observed on the surface of the CSW, indicating that it had a chemical bonding force with the matrix. The CSW was in a straight state with a small deformation capacity, and its prismatic structure increased the mechanical bite force with the matrix. The CSW was observed to be exposed at one end of the matrix surface and appeared broken, indicating that CSW dissipates energy through its own breaking and pulling out [64]. Therefore, CSW enhances the macroscopic performance of the matrix through the mechanical bite force, bonding force, frictional force with the matrix, and its own breaking and pull-out energy dissipation. As shown in Figure 8f, when excessive CSW was added to the matrix, the CSW tended to cluster in regions within the matrix, leading to a decreased compactness of the matrix and reduced macroscopic performance.

From Figure 8g, it can be seen that the BF was distributed in a straight state in the matrix, indicating that BF has a small deformation capacity. The relatively intact BF and the pits left after being pulled out were observed, indicating that BF mainly dissipates energy through pulling out. The observation of pits left by the pulled-out BF being close to the adjacent BF indicates that flaky BF was unevenly dispersed in the matrix. As shown in Figure 8h, there were a small number of hydration product particles on the surface of the BF, indicating the presence of a chemical bonding force between the BF and the matrix and showing that BF is well bonded to the matrix. The slight scratches on the BF surface indicate that relative displacement between BF and the matrix generates frictional force [65]. Therefore, BF enhances the macroscopic performance of the matrix through the bonding force, frictional force, and energy dissipation from its own pulling out.

In summary, when a CSW, AF, and BF hybrid mortar is damaged by stress, the CSW, AF, and BF work together to prevent the development of cracks mainly through the bonding force, friction force, mechanical bite force, and their own pull out and fracture dissipation energy with the matrix interface, so as to improve the mechanical properties and wear resistance of hybrid fiber mortars.

### 3.6. Pore Structure

The pore structure of materials affects their macroscopic performance. Academician Wu classified the pores of cement-based materials into four categories based on their influence on performance: 0–20 nm pores are harmless, 20–50 nm pores are less harmful, 50–200 nm pores are harmful, and pores larger than 200 nm are more harmful [66]. Since mortars with a low water–cement ratio are relatively dense and contain smaller pores, and the nitrogen-adsorption method accurately measures pore sizes below 100 nm, this paper uses the nitrogen-adsorption method to analyze the pore structure of the CAB3 and CAB11 groups with excellent mechanical properties.

**Figure 9:**
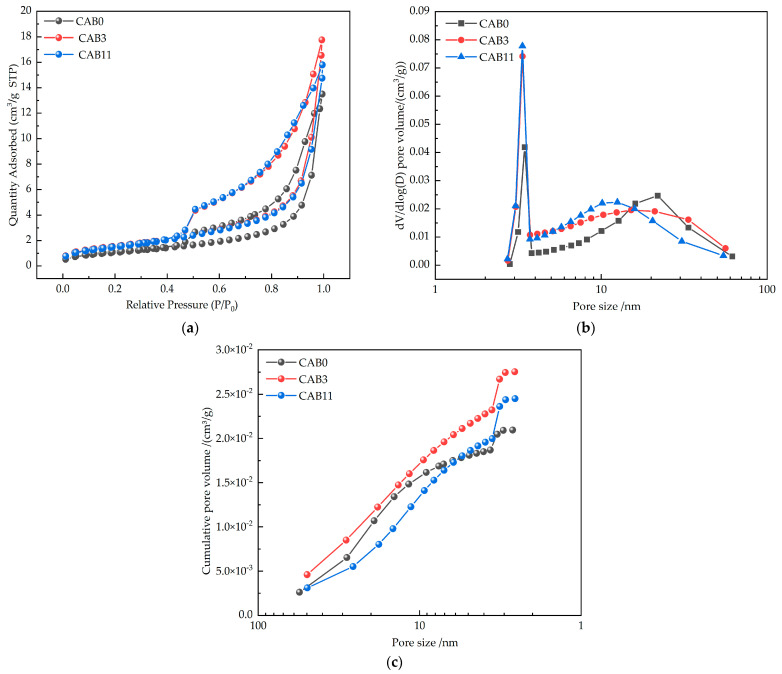
Nitrogen-adsorption pore-size curves of different types of fiber mortars. (**a**) Nitrogen-adsorption isotherms; (**b**) pore-size distribution; (**c**) cumulative pore diameter.

As shown in Table 7 and Figure 9a,b, the most probable pore size of the mortar with 3% CSW (CAB3) and the mortar with 2% CSW + 0.1% AF (CAB11) were the same as that of the control group (CAB0), all located within the gel pore range, but their corresponding pore contents were higher than that of the control group (CAB0). This indicates that the incorporation of 3% CSW and 2% CSW + 0.1% AF has little effect on the most probable pore size range of mortars. Compared with the CAB0 control group, the most probable pore size content of the mortar with 3% CSW and the mortar with 2% CSW + 0.1% AF increased by 76.9% and 85.7%, respectively, indicating that the incorporation of 3% CSW and 2% CSW + 0.1% AF can significantly increase the most probable pore size content of mortars.

As shown in Table 7 and Figure 9a,c, the harmful pore volume of the mortar with 3% CSW (CAB3) was smaller than that of the control group (CAB0), while the less harmful pore volume and harmless pore volume were larger than those of the control group (CAB0), and the average pore size was also smaller than that of the control group (CAB0). This indicates that the incorporation of 3% CSW can refine the pore structure of mortars, reduce harmful pores, and increase less harmful pores and harmless pores, thereby improving the mechanical properties of mortars. The mortar with 2% CSW + 0.1% AF (CAB11) also had similar effects, with its harmful pore volume being smaller than that of the control group (CAB0), while the less harmful pore volume and harmless pore volume were larger than those of the control group (CAB0), and the average pore size was also smaller than that of the control group (CAB0). This indicates that the incorporation of 2% CSW + 0.1% AF can also refine the pore structure of mortars, reduce harmful pores, and increase less harmful pores and harmless pores, making the mortar structure denser. Therefore, CAB11 exhibits excellent mechanical properties macroscopically. Li [67] also confirmed that the incorporation of aramid fibers can refine the pore structure. Wan [23] also found that calcium sulfate whiskers can optimize the pore size distribution. In summary, a proper amount of AF and CSW can reduce and refine the pore structure.

### 3.7. Mechanism of Wear-Resistance Enhancement

**Figure 10:**
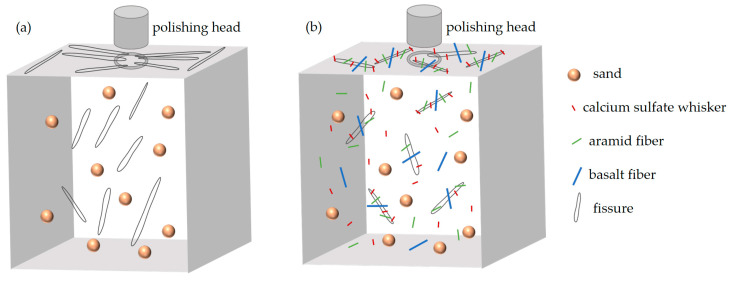
Schematic diagram of the mechanism of improved abrasion resistance by hybrid fibers.

As shown in Figure 10a, when the rotating ball under normal pressure contacted the mortar surface, it caused friction on the mortar specimen surface, leading to adhesive wear. The ball adhered to and peeled off part of the material from the specimen surface, causing damage to the mortar surface. During the wear process, some particles remained on the mortar surface. With changes in the friction force and repeated actions, these particles were pressed back into the mortar surface or rolled between the grinding head and the specimen surface, causing shearing and plowing actions [68]. Eventually, due to repeated stress changes, the wear process on the specimen surface intensified, leading to a fatigue wear failure of the specimen.

As shown in Figure 10b, the three different types and sizes of the fibers CSW, AF, and BF interweaved in the mortar, forming a random three-dimensional network support system, reducing the bleeding and segregation of the mortar, increasing the bonding force between the cementitious material and the aggregate, and increasing the compactness of the mortar structure, thereby improving the wear resistance of the mortar. At different structural and performance levels of the mortar and at different stress stages, these fibers sequentially reinforced and toughened the mortar, fully utilizing their respective size and performance effects, reducing and narrowing microcracks in the mortar, and alleviating stress at the crack tips, thereby further enhancing the wear resistance of the mortar [69]. Additionally, CSW, AF, and BF can optimize the pore structure of a mortar, increasing its density, thereby further enhancing the wear resistance of the mortar [10,70,71]. When part of the hardened paste was worn away, the exposed hybrid fibers could still bear the friction load, delaying the wear failure process of the mortar. In summary, hybrid fibers mainly enhance the wear resistance of mortars by improving their density, preventing cracks, and bearing friction loads.

## 4. Conclusions

In this paper, the effects of a combination of AF, CSW, and BF on the compressive strength, flexural strength, and wear resistance of cement mortar composites with a low water–binder ratio were studied. The microstructure and strengthening mechanism were studied by means of SEM and BET. The main conclusions are as follows:

(1) The results of the mechanical tests show that hybrid fibers significantly enhance the compressive strength and flexural strength of mortars. Among them, the mortar with 2% CSW + 0.1% AF had the highest compressive strength, while the mortar with 4% CSW + 0.067% AF + 0.033% BF had the highest flexural strength.

(2) Hybrid fibers also significantly improve the wear resistance of mortars. The wear-resistance test results show that the mortar with 2% CSW + 0.1% AF had the best wear resistance. The wear resistance of different types of fiber mortars does not directly depend on their compressive strength grade; compressive strength is just a related indicator of wear resistance.

(3) CSW, AF, and BF mainly dissipate energy through the bonding force, friction, mechanical bite force with the mortar matrix interface and their own pull out or breakage, thereby enhancing the mechanical performance and wear resistance of the hybrid fiber mortar.

(4) The incorporation of 3% CSW and 2% CSW + 0.1% AF can refine the pore structure of mortars, reduce the content of harmful pores, and increase the number of less harmful pores and harmless pores, thereby improving the mechanical properties of mortars.

## Figures and Tables

**Table 1:** Chemical compositions of cement, silica fume, and fly ash.

Raw Materials	SiO_2_	Al_2_O_3_	CaO	Fe_2_O_3_	SO_3_	MgO	K_2_O	Others
Cement (%)	20.34	4.4	62.92	3.2	2.8	1.78	0.45	0.3
Silica Fume (%)	88%	1.63	0.4	1.15	0.05	0.8	0.49	3.21
Fly Ash (%)	48.62	35.41	6.61	6.26	0.63	0.58	0.91	1.25

**Table 2:** Elemental composition of fiber surfaces.

Raw Materials	O	Si	S	Ca	C	Al	N
AF (%)	15.95	-	-	-	79.43	-	4.62
BF (%)	58.93	26.43	0.59	4.93		9.12	
CSW (%)	30.27		27.56	40.46	1.71		

**Table 3:** Physical parameters of calcium sulfate whiskers.

Fiber	Single Filament Diameter (μm)	Length (um)	Bulk Density (g·cm^−3^)	Absolute Density (g·cm^−3^)	PH Value
Calcium Sulfate Whiskers	1–4	10–200	≤0.5	2.69	6.5–7.5

**Table 4:** Physical parameters of aramid fiber and basalt fiber.

Fiber	Single Filament Diameter (μm)	Length (mm)	Specific Gravity (g·cm^−3^)	Tensile Strength (MPa)	Elastic Modulus (GPa)
Aramid Fiber	12	6	1.68	2880	94
Basalt Fiber	15	12	2.64	2070	84.2

**Table 5:** Mix design for the mortar control group.

Parameter	Water (kg·m^−3^)	Fly Ash (kg·m^−3^)	Silica Fume (kg·m^−3^)	Cement (kg·m^−3^)	Sand (kg·m^−3^)	Water-Reducing Agent (kg·m^−3^)
Value	180	90	63	747	890	9

**Table 6:** Experimental scheme for hybrid fiber cement mortar composites with low water-to-binder ratio.

Specimen Number	AF and BF Combination Ratio	Fiber Content (%)			Specimen Number	AF and BF Combination Ratio	Fiber Content (%)		
		AF	BF	CSW			AF	BF	CSW
CAB0	-	0	0	0.00	CAB11	-	0.100	0	2.00
CAB1	-	0	0	1.00	CAB12	-	0.100	0	3.00
CAB2	-	0	0	2.00	CAB13	-	0.100	0	4.00
CAB3	-	0	0	3.00	CAB14	-	0	0.100	1.00
CAB4	-	0	0	4.00	CAB15	-	0	0.100	2.00
CAB5	-	0.100	0	0.00	CAB16	-	0	0.100	3.00
CAB6	-	0	0.100	0	CAB17	-	0	0.100	4.00
CAB7	2:1	0.067	0.033	0	CAB18	2:1	0.067	0.033	1.00
CAB8	1:1	0.050	0.050	0	CAB19	2:1	0.067	0.033	2.00
CAB9	1:2	0.033	0.067	0	CAB20	2:1	0.067	0.033	3.00
CAB10	-	0.100	0	1.00	CAB21	2:1	0.067	0.033	4.00

Note: AF stands for aramid fiber; BF stands for basalt fiber; CSW stands for calcium sulfate whisker.

**Table 7:** Nitrogen-adsorption test results of different mortars.

Specimen Number	Average Pore Diameter (nm)	Most Probable Pore Diameter (mL/g)	Harmless Pore Volume (mL/g)	Slightly Harmful Pore Volume (mL/g)	Harmful Pore Volume (mL/g)
CAB0	11.2914	0.0419	0.26035	0.00652	0.00261
CAB3	8.9263	0.07412	0.31199	0.01308	-
CAB11	7.8503	0.07781	0.261	0.00862	-

## Data Availability

Due to the participants in this study not agreeing to disclose the data, there are no publicly available data.
